# Warning, or Manipulating in Pandemic Times? A Critical and Contrastive Analysis of Official Discourse Through the English and Spanish News

**DOI:** 10.1007/s11196-021-09869-z

**Published:** 2021-11-13

**Authors:** María Ángeles Orts, Chelo Vargas-Sierra

**Affiliations:** 1 Department of Translation and Interpreting, University of Murcia, Plaza de la Universidad, s/n., 30001 Murcia, Spain; 2 Department of English Studies, University of Alicante, Carretera de San Vicente del Raspeig, s/n., 03690 San Vicente del Raspeig, Spain

**Keywords:** Critical Discourse Analysis, Political persuasion, Covid19, Lexical manipulation, Power in discourse, Emotional implicature

## Abstract

Focusing on media discourse and adopting a Critical Discourse Analysis—linguistic and rhetorical—perspective, this paper explores the role of the media in influencing citizens’ behaviour towards the COVID-19 crisis. The paper evaluates the set of potentially persuasive lexical items and emotional implicatures used by two quality newspapers, i.e. *The Guardian* (UK edition) and *El País* (Spain edition), to report on the pandemic during the three waves—the periods between the onset and trough of virus contamination—that occurred until March 2021. A representative, ad-hoc, comparable corpus (COVIDWave_EN and COVIDWave_ES) was compiled in English and Spanish comprising the news on the pandemic that appeared in the aforementioned newspapers during the three established time periods. The corpora were uploaded to Sketch Engine, which was used to first detect and analyse different categories (nouns, verbs, and adjectives) of word frequency, and then assign negative or positive polarity. Lexical keyness was secondly analysed to categorize emotional implicatures of control, metaphors, signals of epistemic asymmetry and positive implicatures in order to discern how they become weapons of negative or positive persuasion. The ultimate end of the study was to critically analyse and contrast the lexicon and rhetoric used by these two newspapers during this time period so as to unveil the stance taken by governments and health institutions—voices of authority—to disseminate words of control and persuasion with the aim of exerting influence on the behaviour of citizens in UK and Spain.

**Supplementary Information:**

The online version contains supplementary material available at 10.1007/s11196-021-09869-z.

## Introduction

The study here presented constitutes a contrastive lexical and rhetorical analysis of a corpus of news items collected around the COVID 19 pandemic from two examples of the so-called quality press in the United Kingdom and in Spain: The British *The Guardian* and the Spanish newspaper *El País*, during the months of March 2020 to March 2021. This is, hence, a diachronic study, since it deals with the language of control and persuasion used during the course of the different outbreaks of the disease and their impact on society, which have been described as the first, second and third ‘waves’ of the infectious process.

The first premise of our work is that both publications, as prestigious newspapers, constitute a credible voice for establishing and developing public opinion “as the national will” [1] in a context as serious and disastrous as a pandemic. This implies that there will be some kind of collusion between these prestigious media and governments, for, as [2] point out, a crisis poses threats, generates deep uncertainty and puts the flexibility of elites to the test in the face of fast-moving and highly intertwined challenges. Governments, therefore, need to rely on prestige media as instruments providing certainty in the face of threatening situations, amidst the information morass of today’s digital media and/or social networks. Generally speaking, the media is seldom unbiased, leaning towards a specific viewpoint that might support or oppose government policy, in tune with the ideology of their readers or their own editorial line; however, the dependency between prestige media and official sources must necessarily be enhanced in a natural disaster such as a pandemic [3: p.1–2], a context of crisis like few others, in the course of which institutions must propagate and implement forceful and urgent patterns of action so that the climate of uncertainty does not prolong the crisis over time, or increase the level of insecurity.

The second premise of this study, which results from the first, is that the pandemic has led to a stern suspension of freedoms on those that Wagner et al. have called *the third front line,* i.e., “the population on which the government imposed severe restrictions on movement, except for priority reasons” [4: p. 2]^1^. Hence, governments and official think tanks with the aid of the media, had “an invisible control over citizens’ bodies, within either the public sphere or the private domain”, strongly, but subtly, exerting their influence upon those citizens to monitor their movements and guide behaviour. As we intimate above, power was seldom, in this case, exerted coercively, by physical force, but was deployed through ‘pastoral’ reasons, authorities making use of their position of domination on those “of lower status” through the perception that the former were in charge of providing “protection, aid and support” [5: p. 32].

The third premise—and our main work hypothesis—also stems from the previous two; it addresses both the nature of the language of authority and persuasion deployed by the media, and the analytical perspective of this work, which is situated in the field of Critical Discourse Analysis (CDA). CDA is interesting to Semiotic studies because it adds a sociological dimension to Linguistics and orthodox Discourse Analysis. It explains how writers and speakers achieve, maintain, and reproduce social power and manipulation through discourse. Analysing the discursive strategies deployed to legitimately control or ‘naturalize’ the social order, CDA considers the opaque processes of domination through language, explaining how it constitutes a powerful social tool at the service of the powerful. In their theses, the relationship between discourse and power dominance is achieved by reproducing and maintaining core asymmetrical relations between addresser and addressee [6–9].

In order to exercise control over the population, governments (in this case, both the British and the Spanish) acted by imposing their hierarchical superiority through various legal instruments widely disseminated by the mass media, but—and mainly–2, they have also done so ‘symbolically’ [10: p. 89]: projecting their discourse of authority through the press in the use of (a) both negatively and positively-polarized persuasion and (b) of very specialized, very technical information that revealed a pronounced epistemic asymmetry between the issuers (‘the experts’, in this case, governments, through the mass media) and the receivers (‘the non-experts’, or laypeople, represented by citizens at large) of texts [11]. In doing so, the State took over the knowledge resources of health authorities allegedly for the common good, but also to retain what Gramsci [12] called ‘cultural hegemony’ and Bourdieu named ‘symbolic violence’ [13], i.e., the justification of the social, political, and economic *status quo* of the dominant as natural and inevitable, perpetual, and beneficial for everyone. Hence, both negatively-polarized emotional language—to persuade through fear and (negative) anticipation, as the two basic emotions arising from hierarchical relationships [Plutchik op. cit. 6: p. 24]—and specialized language—to achieve distance from addressers and receivers of the message—were exhibited as instruments of power to implicitly establish control and imposition on the part of authorities.

On the other hand, because of the need to legitimize their discourse of power, and even if there is no persuasive language such as the language of authority [14: p. 50], consent was needed from the citizens to agree to new—and incredibly harsh—norms and regulations, such as self-isolation, quarantining and the use of face masks or coverings that distorted any representation of everyday normality. Thus, inasmuch as the government through the press is the dominant ‘discursive community’ [15] in times of crisis, it needs to work towards consensus, which is achieved through legitimation as the process of ‘explaining’ and ‘justifying’ [16: p. 92–95] to gain credibility.

And because especially during the three first waves of the COVID crisis legitimation needed such credibility, it could not be attained through imposition, but through the deployment of techniques of positively-polarized verbal persuasion to convey the suitability of prosocial interventions triggering positive sentiment and emotional engagement to attain message efficacy and greater compliance [17]. The way in which both impositive and persuasive language occur in our corpus (in either language, and in each of the three waves) is precisely the matter of our work.

## Negative and Positive Persuasion, as Products of Agonic and Hedonic Societies

The tension between negative and positive persuasion, the duality domination-manipulation and legitimation, is clearly explained in the light of socio-evolutionary theory of emotions [5, 18]. In fact, it is our view that these two ways to persuade connect with two aspects in which humankind resolves the main existential problems it faces during its evolution: agonic and hedonic societies.

According to the ethologist Chance [op.cit. 6: p. 42], agonic and hedonic societies are the two ways to contemplate the interactions we maintain within society, which are, in turn, and as TenHouten explains [5: p. 42–46], related to Alan Fiske’s [19] four relational models of society, or four human modes of relationship, i.e. ‘authority-ranking’ (arrangement into a hierarchy), ‘communal sharing’ (having things in common), ‘equality-matching’ (striving to maintain egalitarian relationships) and ‘market-pricing’ (use of ratios).

From TenHouten’s point of view, hierarchy and market-pricing pertain to the dominion of agonic societies, “competitive, conflictual and hierarchically organized around the concept of social dominance” [5: p. 43], which implies that both individuals and communities end to establish egotistical and antagonistic relationships to maintain their status and control resources. This would be the area that we have decided to cover through the analysis of negative persuasion: words of control, epistemic asymmetry and manipulative rhetorical mechanisms.

Epistemic asymmetry, specifically, happens when there is a very specialized community interacting with non-experts and entails a monopolistic usage of gnoseological resources. It is our assumption that the discourse of public health was deployed by the State in an exercise of ‘interdiscursive appropriation’ [20] to provide the executive with rightful, well-documented, powers to manage the citizens’ fate for their own good. Contrarily, equality-matching, and communal sharing form the basis of informal, hedonic communities, which hinge around the concepts of human altruism, social cooperation and support and reciprocity. The need to establish shared values, for individuals to identify themselves into a wider collective is fundamentally emotion-based. This perception of identity and communal belonging (the notion of ‘sameness’, versus the notion of ‘otherness’) is central in world politics since it explains the way in which we attach and situate ourselves in the social world [21]. More importantly, emotions play a key role in achieving communal cohesion in the face of a crisis such as the COVID19 pandemic. As Bleiker and Hutchinson [21: p. 130] state:

Emotions play a central role at all times: they lie at the heart of how communities, including states, are organised and function. But traumatic events challenge and often uproot related attachments, exposing their emotional nature in a particularly acute and visible manner.

Heffner et al. [17: p.1] affirm that both the instilment of fear and prosocial engagement have played a role in the C19 crisis. Since the virus can only be slowed by extreme behavioural changes in conduct and societal coordination, emotional engagement through fear or the need to obtain societal and communal benefits has been a critical component of behaviour change.

As Iqbal et al. [22: p. 211] point out, political leaders intentionally use different linguistic and persuasive skills to impose their ideologies on the population, which happily accepts them. Indeed, as has been anticipated in our introduction, political persuasion as ‘successful public communication’ [10: p. xviii] needed to be used during the first, second and third waves of the COVID19 pandemic to supervise and exert control over the population. Disregarding here other models of Aristotelian public rhetoric^3^, there are, according to our own perspective—and as has been intimated by now–, two ways in which persuasion may occur in political language: with a negative and with a positive polarity.

‘Negative persuasion’ happens in the context of an agonic society, i.e., when authority and manipulation are deployed through an array of methods to coerce individuals to abide by the rules. It is what Weber defined as “the potential of one person to impose his will on another, irrespective of the other person’s desires and resistance” [Weber op. cit. 23: p. 1]. But imposition and manipulation are not the same thing: in our view, the former happens when threatening and language of negative anticipation (mostly, in the shape of negatively-polarized words of fear and command, and deontic modals) are used as “the enabling mechanisms for the domination, coercion and control of subordinate groups” [24: p.3]; the latter happens, in contrast, when more opaque linguistic processes produce a specific type of automatic and thoughtless compliance on the part of individuals; the willingness to say ‘yes’ without thinking first [25: p. 73].

We have studied manipulation mainly through negative emotional implicatures, called ‘e-implicatures’ [26], invoking fear and control. In our corpus, as we shall see, these occur when emotions are not expressed explicitly, but invoked through lexicon with a negatively or positively emotional connotation (and not denotation). But we shall study other manipulative devices to curtail the freedom of individuals which are also implicatures of sorts: epistemic asymmetry and ontological or propositional metaphors [27] reifying institutions. With epistemic asymmetry, citizens are showered with all kinds of technolects and acronyms that leave them in awe of expert wisdom on the part of authorities; with ontological metaphors, the Government, the State or the health authorities are granted the stature of human beings with the unitary power to command and discipline the population. Positive persuasion happens, on the other hand, with a hedonic orientation: when the discourse of power needs to be legitimized, thus becoming the discourse of society at large. In our study, we have considered that this kind of persuasion is deployed through positive-polarity emotion words and e-implicatures—peculiar to the COVID19 discourse, as we shall see below in the course of the study–, to persuade citizens that the rules applied to them are geared towards the common good and the optimum organization of society.

We are using the Attitude paradigm within Appraisal Theory [28–30], specifically the subparadigm of Affect, which indicates positive assessment as an emotional reaction in three main types: happiness, security, and satisfaction. Attitude explains how writers express emotional points of view, pass judgment on people and/or on the aesthetic quality of a process, phenomenon, or text. These evaluations or emotional responses reinforce (explicitly or implicitly, as happens with positive e-implicatures) the solidarity between the sender and his audience and create a bond between writer and reader. These words are akin to what Whyte [op. cit. 16: p. 49] calls ‘hooray’ (as opposed to ‘boo’) words, that is to say, those words are deployed in politics to exploit their positive connotation. They convey the feeling that individuals obliged to follow harsh rules must feel they have the power to follow such rules or not and are not at the mercy of a blind and arbitrary authority [27]. To which extent these opposing forces happen in our corpus—the need to impose the discursive supremacy of the institutions in power to retain control upon citizens by means of threatening and manipulative language, and the necessity to establish interpersonal relations between their members and those of society at large in the search for group cohesion so as to fight the virus—is what we will discuss throughout our work.

## Material and Methods

A representative, ad-hoc, and comparable corpus has been compiled in English (COVIDWave_EN) and Spanish (COVIDWave_ES) comprising the news on the pandemic that appeared in two quality newspapers, i.e. *The Guardian* (UK edition) and *El País* (Spain edition) during the three time periods according to the COVID-19 data by John Hopkins University 4, as shown in the table:

Three sub-corpora have been subsequently built representing the time interval for each wave in the respective country and word lists have been generated from the whole corpora (each sub-corpus to detect and analyse evaluative and potentially persuasive lexical items by means of different Sketch Engine features, like Keywords, Concordance, Word List and Thesaurus). The choice of the papers was made on the basis of representativeness: their leading role in opinion formation in their country of origin [31: p. 92]. COVIDWave_EN contains 1,948 news-items—including, as we mentioned above, op-eds and editorials–, gathered during the periods shown in Tables 1 and 2. Through the Factiva database we had access to the news-items in both newspapers.

**Table 1:** Time intervals for each wave

	Spain	United Kingdom
1st wave (1stW)	10/03/2020–14/04/2020	25/03/2020–23/05/2020
2nd wave (2ndW)	16/07/2020–13/11/2020	22/09/2020–19/11/2020
3rd wave (3rdW)	15/12/2020–05/03/2021	08/12/2020–02/03/2021

**Table 2:** General statistics for the Spanish and English corpora and sub-corpora

	Whole Corpus		1stW		2ndW		3rdW	
	ES	EN	ES	EN	ES	EN	ES	EN
Tokens	963,212	1,807,845	280,236	364,657	364,881	477,991	269,271	224,459
Words	837,772	1,574,108	245,001	317,268	293,885	415,257	216,010	195,907
Sentences	30,871	64,967	9,662	13,535	10,082	17,258	7,164	7,665
Types	37,671	44,834	18,445	20,463	20,54	23,234	18,132	15,511
TTR	3.86	2.12	6.58	5.61	5.62	4.86	6.73	6.91
STTR	43.26	45.68	44.01	45.37	43.11	45.9	42.62	45.90

To refine the results, we established as filters: (a) the editions of each newspaper (UK and Spain); (b) the language; (c) the keywords to be searched (coronavirus or pandemic or covid-19): (d) Political/General News section; and (e) Domestic Politics as subject. Each news-item was saved as a file, so the English corpus contains 1,948 of those. The same search criteria were used to compile COVIDWave_EN. Our Spanish corpus is, therefore, comparable in terms of content, although we obtained a smaller sample, i.e., we downloaded 1,374 files, hence we obtained almost 500 fewer news items, resulting in a difference of about one million words.

The following table shows the main characteristics of each corpus and sub-corpus:

As the previous table displays, tokens, words, sentences, types, type/token ratio (TTR) and standardized TTR have been calculated. ‘Tokens’ are the smallest units in a corpus and include word forms, punctuation marks, digits, abbreviations, and anything else between blank spaces. That is the reason why a corpus normally contains more tokens than words, since they are a type of token; more specifically, a word is a token beginning with a letter of the alphabet. The term ‘type’ refers to the number of distinct words in a corpus; each is counted only once even if it appears in the corpus several times. TTR is expressed as a percentage and is obtained by dividing the total number of types by the total number of tokens. The higher the value, the more different words the corpus contains. Conversely, a low value indicates a high number of repetitions, which could mean that the corpus is less rich or varied from a vocabulary point of view. It, then, serves as an indicator of lexical diversity or density.

However, the comparison of TTRs between different corpora serves as a reference only when contrasting corpora of similar size, since TTR varies according to the size of the corpus. A larger corpus gives rise to more repetitions and hence its value may be lower. Standardized TTR (STTR) calculates TTR at regular intervals and is used to neutralize the influence of a corpus size when calculating TTR, as larger corpora have more repetitions and, consequently, have lower values than smaller ones. Normalized TTR does not consider word repetition, resulting in a higher average value. As noted above, the English corpus is larger (with the exception of the third wave of the Spanish corpus, with 269,271 tokens), being therefore slightly denser in lexis, more abundant in tokens, sentences and types; in contrast, STTR calculation shows that fewer words are repeated, making it moderately richer in word variety overall. This is an important datum, for the calculation shows that both corpora are quite comparable in terms of lexis, and this study aims to search for persuasion devices mainly through a lexical study of both corpora.

As far as our analysis is concerned, then, the aim was to find traces of negative and positive persuasion in such corpora, indicating the intrusion of the voices of the media as interlocutors of the official voices and think tanks. With that aim in mind, we basically concentrated upon the following two lexical strands of research, i.e., negative persuasion as a force of explicit or implicit coercion, and positive persuasion, as a means for the State to achieve legitimation in their manoeuvres to control the virus in the face of citizens. We have explained above that negative persuasion occurs through bare, undisguised verbal mechanisms, and through manipulation, as the thwarting of *ethos* on the part of the issuer, as has been already analysed in pandemic by Iqbal et al. [22: p. 219–222]. Examples of the first such are the following:

1. Use of verbs in deontic expression, implying the existence of a more powerful issuer in an unequal status relationship in the shape of directives of obligation (‘must’, *deber*), moral obligation (‘should’, *debería*, ‘need to’, *tener que/necesitar*) and /or prohibition or moral impediment (‘must not’, *no deber*, and ‘should not’, *no debería*, respectively)  [Trosborg, op.cit. 35].
2. Use of lexicon indicating control and imposition, mainly nouns of prohibition, obligation, but also those related to legal authority and its application, specifically those to do with police forces and punishments granted by the State to transgressors, as the examples in Table 3:
  Secondly, the examples of manipulation that have been researched in the corpora follow these lines, as intimated above:
3. Emotional implicatures of control, authority, threat; expressions with negative polarity having to do with the containment of the virus, and the measures taken by the State, sometimes similar to warfare images (such as ‘emergency status’ or ‘curfew’), or lexical metaphors having to do with space restrictions (‘jail’ metaphors), such as ‘lockdown’, ‘social distancing’ or ‘phased opening’.
4. Ontological metaphors: when institutions such as the law, the government, or any State institution, including the Social Security (the NHS, in Britain), is granted the status of an animate entity to exert power.
5. Covid-19 technolects: Deployment of very technical words around the disease, technicalities of medicine or logistics similar to the terminology provided by the WHO, but also by epidemiologists, the health authorities and medical journals (‘the experts’) showing epistemic asymmetry.

**Table 3:** Words of prohibition, obligation, and legal control

	English (EN)	Spanish (ES)
Obligation and prohibition	Obligation, order, prohibition, impediment, duty, need	Obligación, veto, impedimento, prohibición, deber
Legal authority	Law, regulation, norm, decree, police, punishment, jail, fine, measure	Ley, reglamento, normativa, decreto, policía, guardia civil, castigo, cárcel, multa, medida

The instruments of manipulation are exemplified in Table 4:

**Table 4:** Words of manipulation: control, power, and epistemic asymmetry

	EN	ES
Emotional implicatures of control	Social distancing, isolation, containment, quarantine, emergency status, lockdown, de-escalation, flat the curve, phased opening, curfew	Distanciamiento social, aislamiento, cuarentena, cierre de emergencia, confinamiento, desescalada, aplanar la curva, toque de queda
Ontological metaphors	the State, the Government, the NHS, the Law	El Estado, el Gobierno, la Seguridad Social, el estado de derecho
Covid 19 technolects	SARS-CoV-2, comorbidity, immunosuppression, PPE (Gear), ventilator, outbreak, variant, outbreak hotspot, chest imaging, chest (CT) scan, fomites, strain, mutation, reservoir host	SARS–CoV-2, comorbilidad, inmunodepresión, EPI o EPP, respirador, ventilador, brote, variante, foco del brote, imagen de tórax, fómites, cepa, mutación, hospedador reservorio

Finally, positive manipulation is used when there is a need to establish common values between issuer and receiver; in other words, when there exists an approximation between both parties—the State through the media, and the citizen–, which takes place with several rhetorical mechanisms, mainly with epistemic verbs reducing the distance between issuers and receivers, explicitly emotive lexical items employed to signal the narrator’s emotional stance and e-implicatures triggering positive emotions, such as the following:

1. Deployment of discretionary verbs and expressions, indicating lack of power distance between the parties, and verbs with a positive connotation, as in Table 5:
2. Use of words with positive polarity from the Affect paradigm of as in Table 6.
3. Use of positive e-implicatures, as well as metaphors with positive polarity, having to do with the defeat of the virus and/or the measures taken to alleviate it, such as digital techniques for home schooling, restoration of normal living or measures to improve the social panorama.

**Table 5:** Epistemic verbs of discretion and permission

	EN	ES
Neutral power distance (prerogatives)	Can, may, agree, accept, allow, grant, consent, acknowledge	Poder, permitir, aceptar, conceder, acceder, aprobar, asentir, ratificar

**Table 6:** Emotion states within the Affect paradigm

Paradigm of affect		Nouns	Adjectives
Happiness cheer, affection	EN	Happiness, cheerfulness, excitement, joy	Happy, cheerful, gay
	ES	Felicidad, alegría, regocijo	Feliz, alegre, alborozado
Security confidence/trust	EN	Confidence, trust, hope, reliance, belief, security, protection	Safe, secure, clear, protective
	ES	Confianza, esperanza, dependencia, convicción, seguridad, protección	Seguro, claro, cierto, protector
Satisfaction interest/pleasure	EN	Satisfaction, contentment, interest, pleasure, gratification, fulfilment	Satisfied, good, better, right, agreeable, interesting
	ES	Satisfacción, agrado, interés, placer, bienestar, plenitud	Satisfecho, bueno, mejor, agradable, interesante, pleno

## Results and Discussion

### *Lexical Frequencies: Significant Words with Negative and/or Positive Polarity.*

Our lexical analysis was divided into two methods of detection. On the one hand, we relied on a lexical extraction technique known as “keyness analysis” [32] to single out the most relevant words in our corpora. This is a quantitative method that involves comparing the frequency lists of two corpora; one being bigger and more general, usually called “reference corpus” (RC), and the other being smaller or more specialized, known as the “target corpus” (TC). The reference corpora used in our research were English Web 2020 (enTenTen20) [33], containing 38 billion words, and Spanish Web 2018 (esTenTen18) [34] with 17.5 billion words, both readily available in Sketch Engine. These corpora contain sub-corpora based on language varieties, so our queries were conducted on these varieties so that the results were equivalent in this respect. For English we used the sub-corpora “UK domain.uk”, with nearly 3 million words, and for Spanish we used the one called “European Spanish domain.es”, containing nearly 9 billion words.^5^

Keyness analysis helped us to gather those implicatures and specific terminology pertaining solely to the crisis, which we eventually translated as emotional implicatures of a negative or positive polarity, as ontological metaphors or as signals of epistemic asymmetry. The analysis of specificity produced by means of Sketch Engine did not allow us, contrarily, to gather the frequency of deontic words, words of control or those with a positive/negative appraisal. That is why we resorted to start our analysis on negative and positive persuasion in our corpora by searching with Sketch Engine for the first 100 nouns, adjectives, and verbs in either corpus, regarding their absolute frequency. The aim was to scan the lexis that predominates in the corpus and systematize it as to its peculiarity (the way the most frequent words picture what the main topics are), or its positive or negative character in either corpus.

We first filtered the results by grouping the most significant substantives into three clusters, i.e., the main characters in the crisis, including political groups, institutions and collectivities, the words for the sanitary crisis, and, finally, the measures taken to stop it or reduce its consequences. Such grouping would, eventually, aid us to comprehend the main arguments in either corpus and ultimately unveil metaphorical mappings when combining them into collocations and n-grams. A subsequent examination of adjectives and verbs would also provide important results as to the peculiarities of the corpus and the difference between corpora.

Tables 7, 8 and 9 summarize our results, as follows:

**Table 7:** Most frequent words in the English corpus

Number/Grouping	Words
38 Main characters (people, institutions)	Government, people, minister, Johnson, country, home, school, England, MP, secretary, NHS, leader, labour, Boris, business, worker, child, party, staff, public, world, job, Hancock, economy, council, state, Britain, downing, family, EU, work, member, BREXIT, company, university, cabinet, committee, community
12 Health crisis	Coronavirus, health, pandemic, covid19, virus, crisis, covid, case, death, risk, infection, concern
20 Measures	Lockdown, care, restriction, test, measure, vaccine, support, plan, rule, testing, system, decision, rate, scheme, response, service, advice, report, adviser, action
47 adjectives	
10 negative	Bad, positive, long, vulnerable, hard, difficult, pandemic, serious, tough, wrong
9 positive	Good, clear, possible, able, right, safe, available, free, protective
14 intensifiers/softeners (10 I, 4 S)	prime, big, great, low, least, important, small, significant, little, essential, necessary, major, intensive, strong
13 neutral	Public, social, local, national, political, economic, British, scientific, medical, European, different, financial, legal
46 Verbs	
18 of action / 5 no action	Do, make, go, take, get, work, follow, face, lead, test, reopen, move, increase, meet, ensure, create, act, seek remain, stay, fail, die, stop
9 of expression	Say, tell, show, announce, speak, write, report, claim, confirm
7 for direct/indirect commands	Need, ask, want, warn, urge, require, force
7 discretionary verbs	Give, help, allow, provide, support, protect, offer

**Table 8:** Most frequent words in the Spanish corpus

Number/Grouping	Words
36 Main characters (people, institutions)	Gobierno, presidente, estado, comunidad, España, Sánchez, país, PP, sanidad, persona, partido, ejecutivo, ministro, Cataluña, ministerio, Generalitat, PSOE, líder, portavoz, Illa, pedro, Vox, Congreso, Govern, Barcelona, oposición, ciudadano, empresa, ERC, Ciudadanos, economía, trabajo, sector, vicepresidente, ayuntamiento, alcalde
10 Health crisis	Pandemia, caso, coronavirus, crisis, situación, alarma, virus, contagio, problema, riesgo
21 Measures	Medida, acuerdo, gestión, plan, reunión, decisión, confinamiento, consejo, consejero, apoyo, ayuda, restricción, sistema, decreto, cierre, ley, prueba, comisión, control, cambio, recurso
43 adjectives	
9 negative	Positivo, peor, duro, difícil, largo, grave, malo, vulnerable, crítico
4 positive	Posible, bueno, mejor, claro
13 intensifiers/softeners (10 I, 3 S)	Grande, necesario, importante, principal, máximo, extraordinario, pleno, esencial, mínimo, pequeño, bajo, fuerte, extremo
17 neutral	
	Sanitario, público, político, social, económico, europeo, catalán, español, autonómico, nacional, central, vasco, laboral, jurídico, judicial, educativo, médico
41 verbs	
16 action / 3 no action	Hacer, poner, cerrar, unir, evitar, tratar (de), salir, defender, prever, trabajar, acabar, suspender, producir, vivir, cumplir, limitar Mantener, fallecer, contagiar
7 of expression	decir, asegurar, explicar, anunciar, afirmar, criticar, informar
10 for direct/indirect commands	Tener (que), deber, pedir, querer, reclamar, insistir, imponer, exigir, obligar, solicitar
5 discretionary verbs	Poder, aprobar, permitir, ofrecer, apoyar

**Table 9:** Words of prohibition, obligation, and legal control

EN Nouns	TC Normalized frequency^a^ (NF)	RC Normalized frequency (NF)	ES Nouns	TC Normalized frequency (NF)	RC Normalized frequency (NF)
*Obligation and prohibition*					
Obligation	21.57	33.81	Obligación	83.06	97.48
Order	248.36	83.74	Veto	43.6	4.66
Prohibition	4.43	4.73	Limitación	112.12	38.9
Constraint	8.85	14.43	Prohibición	48.8	19.56
Duty	82.97	87.23	Deber	32.18	34.34
Need	287.08	279.67			
Liability	13.28	30.93			
*Legal authority*					
Law	252.79	242.6	Ley	413.2	527.59
Regulation	143.26	88.53	Reglamento	38.41	86.11
Norm	6.64	14.03	Normativa	38.41	96.12
Decree	4.98	4.77	Decreto	418.39	103.53
Police	293.72	165.84	Policía	168.19	4.512
Punishment	12.17	13.91	Guardia civil	46.72	39.39
Jail	12.72	7.63	Castigo	14.53	11.24
Fine	63.61	49.81	Cárcel	99.67	25.66
Measure	833.04	95.27	Multa	28.03	26
			Medida	2,121.03	345.31

^a^Because we are comparing frequency between two differently sized corpora, we will always show the normalized frequency (NF). This value is given by Sketch Engine per million words

We will gloss over the results in the following subsections, according to the grammatical category, in turn.

#### Frequent Nouns, and their Implications

Firstly, the classification into three groups of nouns illustrates how the texts on the crisis revolve around similar subjects, with different orders of priority. In the English corpus, political institutions and personae on the one hand (‘government’, ‘minister’, ‘Johnson’, ‘mp’, ‘secretary’, ‘NHS’, ‘leader’, ‘labour’, ‘Boris’, ‘party’, ‘Hancock^6^, and ‘Downing’, for example), are easily distinguishable from the citizens subject to those (‘people’, ‘home’, ‘school’, ‘worker’, ‘child’, ‘family’, where ‘country’, ‘England’ and ‘Britain’ are included). In the Spanish corpus, however, institutions and political parties—*gobierno*, *presidente*, *Sanchez, PP, partido, ejecutivo, ministro, ministerio, Generalitat, PSOE, líder, portavoz, Illa,*^7^*Pedro, Vox, Congreso, Govern*, *ERC, Ciudadanos*, etc.–seem to take precedence over citizens, who are not in the list of most frequent nouns, with the exception of the words *persona* and *ciudadano*. Words like *España* and *país,* pointing to the country as a whole, mix with those referring to a conflictive region, *Cataluña*, and its capital, *Barcelona*, and again suggest political issues that go beyond the management of the crisis itself.

As both tables show, it is the health crisis and its significance which have a central place in both corpora, with almost identical references, where the pandemic and its consequences are dominant: ‘coronavirus’, ‘covid-19’, ‘virus’, ‘pandemic’ (*pandemia*, in Spanish) are ever-present in this group, and in the corpus overall, as well as other words like ‘case’ (*caso*) and ‘risk’ (*riesgo*).

Finally, the actions taken to curtail it are–very negative–lexical metaphors of space restriction, such as ‘lockdown’, ‘restriction’, ‘rule’; *confinamiento* (‘lockdown’), *restricción* (‘restriction’), *cierre* (‘closure’) in Spanish. Some measures with a positive hue occur, however, with ‘vaccine’, ‘support’, ‘service’; *apoyo* (‘support’), *ayuda* (*‘*aid’*)*, *recurso* (‘resource’). Notwithstanding their character, all of them imply the existence of a controlling political machinery in motion, if the Spanish corpus has an added normative character, with words like *ley* (‘law’) and *decreto* (‘decree’), which imply a further degree of prescriptiveness, as corresponds to a highly normative legal system which is the Spanish one, being part of the legislation-based Continental tradition. Along these lines, as we advanced in our previous section, we needed to specifically pursue a search for words of prohibition and control in either corpus, to express negative persuasion, or attempt to coerce by force.

The results shown in this table could not be more interesting. The word *medida* in Spanish occurs mind-bogglingly more often than any other in the table, followed by its English counterpart, ‘measure’. Either word almost multiplies tenfold its appearance in our corpora in comparison to the reference ones, and refer to a kind of oblique negative persuasion, almost an example of an e-implicature of control: they, and their ubiquity, represent a euphemistic sample of how imposition was exerted during the three waves. In fact, every decree (*medida* is followed by *decreto* in appearances, as by-words of the Spanish crisis) or regulation (a word very much in use in the English corpus, by the way) issued by the Spanish government constituted the legal framework to monitor the crisis (as, for example, *Medidas de contención y restricciones para la movilidad*, i.e. ‘Control and Movement Restriction Measures’), in the absence of a comprehensive Act, such as the one passed by the English Parliament, the mention to which (in the word ‘law’) is quite frequent in the English corpus. This could show, again, the heavy reliance that the Spanish legal system has upon normative instruments (as corresponds to a country of Continental, codified tradition), the profusion of which has been especially acute during the pandemic. Passing a sole all-embracing (and very technical) legal instrument is, on the contrary, a signal of the relative lack of frequency of written norms and rules in a country belonging to the Common law system like that of England and Wales, where legislation fulfils a less important role.

Other sources of coercion are present in the mention of the law enforcement forces, the ‘police’, in English, and its Spanish equivalents, *policía* and *guardia civil* (‘civil guard’). Words of obligation are not so striking, but ‘order’ in English and *limitación* in Spanish are marginally outstanding. Additionally, a search was carried out to look for positive nouns in the corpus, or ‘hooray’ nouns, which concord with the Appraisal paradigm.

Table 10 illustrates our findings.

**Table 10:** Positive nouns in the target/reference corpora

EN Nouns	NF (Target corpus)	NF (Reference corpus)	ES Nouns	NF (Target corpus)	NF (Reference corpus)
*Happiness, cheer, affection*					
Happiness	0	12.97	Felicidad	0	24.52
Cheerfulness	0	0.38	Alegría	0	29.73
Joy	7.19	30.11	Entusiasmo	3.11	10.92
Excitement	7.74	15.74	Bienestar	32.18	44.36
Optimism	43.15	5.62	Optimismo	19.73	7.67
Cheering	6.08	2.64	Aplausos	25.95	7.41
Love	31.53	72.22	Amor	3.11	97.06
Loved ones	36.51	5.06	Seres queridos	7.27	2.96
*Security, confidence, trust*					
Confidence	159.31	70.32	Confianza	93.44	70.45
Trust	199.69	58.74	Calma	32.18	11.32
Hope	143.82	66.06	Esperanza	41.53	31.5
Reliance	6.74	6.32	Fiabilidad	11.57	11.95
Belief	40.93	43.65	Convicción	13.1	12.95
Security	156.31	157.83	Seguridad	352.99	260.41
Protection	134.41	117.09	Protección	238.78	166.66
Support	800.4	381.04	Apoyo	464.07	177.92
Determination	17.7	19.32	Determinación	20.76	33.93
*Satisfaction, interest, pleasure*					
Satisfaction	3.87	23.63	Satisfacción	4.15	33.94
Pride	13.28	23.91	Agrado	1.04	4.25
Interest	198.03	258.07	Interés	173.38	272.9
Pleasure	6.08	42.91	Placer	1.04	28.88
Relief	57.53	47.85	Orgullo	6.23	15.03
Fulfilment	2.21	3.57	Plenitud	1.04	4.82

The results are very interesting. Most of the words of the paradigm of Affect appear in negative terms, as compared to their occurrences in the reference corpus. There are some important exceptions, though, mainly in the ‘security’ subparadigm, which we have highlighted in red. These are veritable examples of positive persuasion, ‘hooray words’ to sustain the governments’ work, probably the only ones in the corpus, which we can see where the State, through the press, endeavors to persuade the public to rely on their efforts to save them from the crisis. Words like ‘support’ (the most outstanding of the lot), and its counterpart, *apoyo*, but also ‘confidence’ and *confianza* are very important, as are ‘trust’, ‘hope’, and their variations in Spanish, such as *calma* and *esperanza*.

All of them, if comparatively scarce in the general panorama, summarize the efforts on the part of authorities and media to convince citizens that lack of freedom and obedience will bear their fruits. In the subparadigm of ‘happiness’, ‘optimism’ and *optimismo* also make important appearances, but when seen in context, they normally collocate negatively, as in “caution is needed over bounceback optimism”; “there is too much optimism about post-Covid Britain”, or in Spanish “no hay luga*r por el momento *para el optimismo”. Therefore, with the exceptions regarding adherence to the State’s efforts, the rest of the results are negligible.

#### Adjectives: Negative, Positive and Intensifiers

As we can see in Tables 7 and 8 above, and as far as adjectives go, only the most pertinent were selected, regarding three criteria, namely their meaningfulness within the context of the corpus and its actors, their positive/negative polarity (Tables 7 and 8 above) and their character of intensifiers/softeners. There are striking correspondences in both corpora, since they have a marginally similar number of qualifiers in each one, 47 in the English corpus and 43 in the Spanish one, if the former has almost as many positive results, 11 (‘good’, ‘possible’, ‘able’, ‘right’, ‘safe’, ‘available’, ‘free’ and ‘protective’), as negative, 10, (‘bad’, ‘positive^8^, ‘vulnerable’, ‘hard’, ‘difficult’, ‘long’, ‘pandemic’, ‘serious’, ‘tough’ and ‘wrong’).

Negative polarity predominates in the Spanish corpus, however, with 9 negative results (*positivo*,^9^*peor, duro*, *difícil, largo, grave*, *malo*, *vulnerable* and *crítico*), and only 4 with positive polarity (*posible*, *bueno*, *mejor* and *claro*). Concurrences take place in the ratio intensifiers/softeners, where the former (‘prime’, ‘big’, ‘great’, ‘important’, ‘significant’, ‘essential’, ‘necessary’, ‘major’ and ‘strong’; *grande*, *necesario*, *importante*, *principal*, *máximo*, *extraordinario*, *esencial*, *fuerte*, and *extremo*), are much more usual than the latter in both corpora, undoubtedly stressing the extraordinary circumstances of the crisis, and always happening in negative contexts, such as the following:

1. Social distancing, staying indoors, is really *difficult* for people. It’s particularly *difficult* if you don't have a garden, or if you’re in a flat.
2. It’s *essential* that people who do have the disease are able to be tested *positive.*
3. *Es *necesario* responder al *peor* de los escenarios posibles* (‘It’s imperative to be able to respond to the worst-case scenario’).
4. *Estamos en la primera fase del combate contra el virus. Nos esperan semanas muy *duras*.* (‘We are in the first phase of the fight against the virus. We have some very tough weeks ahead’).

These findings on adjectives overall add up to the general negative, impelling character of the corpora. A further analysis of positive adjectives from the perspective of the Affect paradigm, in contrast, rendered very similar results to the ones obtained for nouns, as in Table 11:

**Table 11:** Positive adjectives in the target/reference corpora

EN ADJ	NF (Target corpus)	NF (Ref. corpus)	ES ADJ	NF (Target corpus)	NF (Ref. corpus)
*Happiness, cheer, affection*					
Happy	66.38	134.88	Optimista	26.99	9.73
Cheerful	5.53	4.72	Feliz	10.38	48.34
Delighted	5.62	4.35	Alegre	0	12.95
Amazing	14.93	77.1	Contento	5.19	24.8
Brilliant	12.72	48.69	Encantado	6.23	34.35
*Security, confidence, trust*					
Safe	289.85	124.42	Seguro	88.25	131.2
Secure	24.34	45.05	Claro	225.29	177.64
Clear	490.09	190.86	Cierto	75.79	160.26
Protective	159.31	15.26	Protector	5.19	13.45
Supportive	18.81	17.96	Solidario	17.65	34.76
Reassuring	11.62	2.99	Tranquilizador	1.08	0.92
*Satisfaction, interest, pleasure*					
Satisfied	2.21	3.32	Satisfecho	4.15	27.75
Good/better/best	914.35	1,416.92	Bueno	362.33	770.69
Right	289.85	184.38	Mejor	238.78	637.06
Pleasant	3.32	23.13	Agradable	3.11	33.44
Interesting	22.13	114.35	Interesante	11.42	109.76
Comprehensive	37.61	49.35	Pleno	188.95	72.97

As we can see, most of the adjectives render negative results, mainly in the subparadigms of ‘happiness’ and ‘satisfaction’ (with the exception of ‘right’, in the latter, with no equivalent or counterpart in Spanish, which mostly refers to orders from the Government for citizens to comply, as in sentences ending in “which is the right thing to do” or “doing the right things to prevent the disease”). In the ‘security’ subparadigm, the only one containing salient results, words like ‘safe’, ‘clear’ and ‘protective’ collocate with words like ‘measure’, ‘instruction’ or ‘procedure', again seeming to instil obedience and respect for the State’s policies. ‘Reassuring’, in turn, often occurs in negative contexts, when the press echoes misgivings on the part of the public opinion, as in “PM's back-to-school talk isn't reassuring for those in fear of Covid-19”.

The only positive adjective in the Spanish sub-corpus is *claro*, in the ‘security’ subparadigm, mostly in collocation, again, with the words *medida* (‘measure’), *estrategia* (‘policy’) or *instrucción* (‘instruction’), mostly pointing to self-assuredness on the part of the State as to their orders to the public.

#### Lexical and Deontic Verbs: Authority and Control in the Corpus

Lexical verbs were categorized into four groups, as shown in Tables 7 and 8 above. The first category is that of verbs of action/no action, if the former is much more habitual (‘do’, ‘make’, ‘create’, ‘ensure’, and *hacer*, *poner*, *evitar*, *defender*, as examples), justifying the activity of the State against the virus. The second group gathers verbs of expression, those implicitly or explicitly invoking an order or a command (and, hence, exerting some degree of hierarchical authority in the sender’s part), such as ‘say/tell’, ‘announce’, ‘claim’ and ‘confirm’, and in Spanish *decir*, *asegurar*, *explicar* and *anunciar*, which also show the dynamicity of the State to disseminate the actions taken to fight the crisis.

These two groups of verbs evoking action or expression would definitely become part of the verbal devices used by the State to legitimize the control exerted over the population, inasmuch as they make its fight against the illness visible. Additionally, and as indicated in our previous section, we searched for the verbs in deontic expression extracted from our corpora along with their normalised frequency (NF), expressed as number of occurrences per million words. We compared these data with the normalized frequency in reference corpora, which were, for the Spanish, esTenTen18, and esTenTen20 for English, as noted before. Both are available in Sketch Engine. This comparison allowed us to see if obligation and prohibition were salient features in COVIDWave_EN and COVIDWave_ES (focus corpora).

Table 12 shows our findings, with highlighting in red for the more salient results:

**Table 12:** Deontic verbal expressions, showing high power distance in the corpora

EN Verbs in deontic expression	NF (target corpus)	NF (ref, corpus)	ES Verbs in deontic expression	NF (target corpus)	NF (ref, corpus)
*Obligation (Highest power distance)*					
Must	636.12	342.32	Tener que	851.32	412.25
Need to	782.7	468.06	Ser obligatorio	25.95	2.26
Should	1,252.32	793.92	Obligar	243.98	127.29
Oblige	19.91	11.6	Haber que	480.68	121.84
Forbid	7.19	6.55	Ordenar	95.51	55.33
Order	93.48	77.11			
*Prohibition (Highest power distance)*					
Must not/musn't	48.12	14.05	No deber	43.6	87.18
Should not/shouldn't	146.58	64.33	Limitar	169.23	53.98
Must be to	2.77	0.74	Prohibir	133.93	43.62
Be not allowed	8.3	7.09	No permitir	84.09	35.93
Deter	9.4	6.06	Impedir	159.88	81.08
			Vetar	17.65	5.06

As we can see, verbs expressing obligation in our corpora show a higher incidence than in the corpora of reference. This impression of urgency and power distance mostly happens in the English corpus, and in the group of obligation, and not prohibition, especially with the verb ‘should’, where the level of incidence almost doubles that of the reference corpus, if the presence of ‘must’ and ‘need to’ is also noticeable.

Nevertheless, in the Spanish corpus, *tener que* and *haber que* (both translatable as ‘have to’) are also remarkably present, as are the verbs of prohibition *limitar* (‘limit’, ‘restrict’), *prohibir* (‘forbid’, strangely quite absent in its English counterpart) and *impedir* (‘prevent from doing’). All the other verbs of imposition are more negligible in appearance, but show some presence in the corpora, demonstrating then that deontic obligation is ubiquitous, then, in both corpora, with more or less hedging strategies.

Finally, verbs exerting positive persuasion or persuasion by legitimation, would be those which constitute prerogatives, conveying power to the receiver of the action—citizens–, such as ‘grant’, ‘consent’, ‘provide’, or, in Spanish, *poder*, *aprobar*, *permitir*, *acceder* or *ratificar*, as gathered in Tables 7 and 8 above*.* Table 13 shows how relevantly some of them appear in our corpus, together with enabling English modal verbs such as ‘can’ and ‘may’, with their relevance as compared to the reference one:

**Table 13:** Discretionary verbs showing prerogative power in the corpora

EN discretionary verbs	NF (target corpus)	NF (ref. corpus)	ES discretionary verbs	NF (target corpus)	NF (ref. corpus)
Can	2,026.72	1.972,61	Poder	3,410.46	3,640.15
May	904.94	657.56	Permitir	596.96	566.4
Acknowledge	92.3	14.73	Aceptar	182.72	117.46
Agree	262.74	176.72	Conceder	62.29	77.56
Accept	147.69	87.42	Acceder	75.79	112.64
Allow	631.69	382.54	Aprobar	605.27	171.25
Grant	169.73	55.97	Asentir	5.19	3
Consent	17.15	5.35	Ratificar	44.64	19.96

At first sight, we can see how Sketch Engine gathers how in the English corpus ‘may’, ‘allow’, ‘acknowledge’ and ‘grant’ are the verbs with the highest relevance, as compared to the reference corpus, and only *aprobar* (‘pass’, ‘sanction’) and *ratificar* (‘ratify’) are salient in the Spanish one. Regarding the latter, they mostly appear in our corpus in the context of ‘ratifying a governmental decision’ (*ratificar una medida del gobierno*) or ‘sanctioning a law’ decree (*sancionar un decreto-ley*), which gives strength to our thesis that the State and its mechanisms (political parties included) are the ones in charge of the conversation in the Spanish context of the crisis.

Even the verb *poder* is, in this corpus, less frequent than in the reference one, which gives an idea of the lack of will granted to the population. All in all, the figures for each discretionary verb are not, by far, as remarkable as the results for the most outstanding verbs of obligation in Table 13.

### Rhetorical Study: Key Words as a Breeding Ground for Implicatures, Epistemic Asymmetry and Ontological Metaphors

A further analysis on keyness, proposed at the beginning of this section, allowed us to search for e-implicatures, metaphorical ontologies and epistemic asymmetries. Tables 14 and 15 present the results of our study, which have been subdivided first according to the data obtained from the general corpus in each language, and to their—first, second, third—subsequent waves. This proves that there are some terms which cropped up only in each of the waves, and not in the general corpus, reflecting that new situations and contingencies called up for new expressions in each phase of the crisis, since neither the State nor the citizens were able to predict what was going to happen next in the face of such unprecedented phenomenon.

**Table 14:** Examples of manipulative devices in the English corpus

Emotional implicatures of control	
SW general	Lockdown, self-isolate, isolation, distancing, test-and-trace, u-turn, quarantine, front-line, restriction, contact-tracing, tracing, austerity, vaccinate, jab, rollout, hospitalization, curfew, anti-lockdown
1st wave	Non-essential, drive-through, repatriation, nrpf, scrutinise, bulk-buy
2nd wave	Firebreak, breaker, mask-wearing, worst-hit, covid-related
3rd wave	JCVI, easing, roadmap (for lifting lockdown), normality, pre-pandemic
MW general	mass testing, herd immunity, social distancing, vaccination programme, coronavirus /first /second /new/ national/full lockdown, circuit breaker, first/second wave, intensive Care,infection wave, physical distancing, death toll, test-and-trace system, economic damage, contact tracing, testing capacity, stay alert, exit strategy, vaccine rollout, first dose, death rate, coronavirus testing, public inquiry, hotel quarantine, scientific advice, nhs test, vaccine programme, transition period, lateral flow, non-essential movement
1st wave	Antibody test, community testing, urgent assistance, national emergency, frontline nhs, current crisis, chequer, cobra meeting, stockpile
2nd wave	Alert level, winter economy plan, four-week lockdown, pandemic response, nationwide lockdown, infected person, three-tier lockdown, moonshot testing
3rd wave	first dose, third national lockdown, red list, third lockdown, travel ban, second dose, International travel, first jab, negative test, nhs test, testing regime, negative result, vaccination centre, vaccine supply
*Ontological metaphors*	
SW general	Downing, tory/tories, dhsc, nhs, thinktank, whitehall, phe, pmqs, dfe, snp, labour, tuc, mhra
1st wave	Rightwing, nasuwt, UK, nhsx, commons, TFL, westminster, nervtag, wuhan,
2nd wave	Merseyside, manchester, covid-19, nao, lancashire, midlands, liverpool, opinium,
3rd wave	SPI-M, frontline, CRG
MW general	NHS staff, scientific advice, coronavirus pandemic, frontline staff, select committee, conservative party, city region, shadow health, european commission, welsh government, new coronavirus, Covid 19 pandemic, ofqual, global pandemic
1st Wave	Advisory group, frontline nhs, virtual parliament, pandemic influenza
2nd Wave	Party conference, supreme court, (former) cabinet, conservative party conference, government source, Scottish government, serco
3rd Wave	Red wall, shadow education, coronavirus crisis, second world, new coronavirus, shadow home, school staff, vaccination centre
*Covid 19 technolects*	
SW general	PPE, ventilator, astrazeneca, vaccine, virus, biontech, hydroxychloroquine, cygnus
1st wave	chloroquine, sars, gown
2nd wave	Moderna
3rd wave	Transmissible, transmissibility, vaccination, vaccinate, pfizer, glp1, ivermectin
MW general	New variant, new strain, coronavirus vaccine, coronavirus response
1st wave	Protective equipment, personal protective equipment, exercise cygnus
2nd wave	Large epidemic
3rd wave	New strain, south african variant, african variant, r number

**Table 15:** Examples of manipulative devices in the Spanish corpus

Emotional implicatures of control	
SW general	Confinamiento, contagio, rebrote, cuarentena, rastreador, pcr, alarma, uci, telemático, mascarilla, confinar, presupuestos, aplazamiento, contagiar, alertas, epidemia, aforo, queda, aplazar, respirador, vacunar, prórroga, asintomático, perimetral, vacunación, parón, restricción, rastreo, pandémico, desescalada
1st wave	Funeraria, extrasociales, desinfección, moratoria, drástico, propagación, emergencias, endurecer, requisar, excepcionalidad, infectar
2nd wave	Ceti, cuarentena, epidemiólogo, temporero, brote, censura, conviviente, aglomeración
3rd wave	Inmunización, crispación, perimetrales, indecisión
MW general	Estado de alarma, crisis del coronavirus, gestión de la pandemia, crisis sanitaria, emergencia sanitaria, declaración del estado, gestión de la crisis, toque de queda, material sanitario, prueba pcr, confinamiento domiciliario, ola de la pandemia, prórroga del estado, conferencia de presidente, cierre perimetral, evolución de la pandemia, inicio de la pandemia, transmisión comunitaria, plan de choque, expansión del coronavirus, coordinación de alerta, situación sanitaria, test rápido, caso de coronavirus, confinamiento total, forma telemática, distanciamiento social, confinamiento perimetral, efecto de la pandemia, situación epidemiológica, decreto de alarma, distancia de seguridad, equipo de protección, curva de contagio, cuidado intensivo, zona de riesgo, medida excepcional, medida de distanciamiento, restricción de movilidad
1st wave	Consejo de ministro extraordinario, hospital de campaña, expansión del virus, efecto del coronavirus, propagación del coronavirus, batalla política, cuidado intensivo, unidad militar de emergencia, pleno telemático, cama de uci, decreto del estado, suspensión de empleo, cumbre europea, cierre de frontera, cierre de colegio, caso positivo, pico de la epidemia, gestión de la residencia, emergencia nacional
2nd wave	restricción social, medida restrictiva, medida sanitaria, aumento de casos, falta de rastreador, riesgo de rebrote, pcr positiva, zona básica de salud, cierre del ocio, radar covid, test serológico
3rd wave	Plan de vacunación, dato epidemiológico, contacto estrecho, test de antígeno, riesgo extremo, protocolo sanitario, presión asistencial, dosis de la vacuna, propagación del virus, fase de vacunación, consecuencia de la pandemia, estrategia de vacunación, ocupación de la uci, actividad diagnóstica
*Ontological metaphors*	
SW general	Pandemia, vox, govern, erc, moncloa, cs, psc, procés, generalitat, ume, sanidad, ciudadanos, esquerra, procicat, ceti, parlament, pdecat, pnv, bildu, gobiernos, cataluña, eurogrupo, compromís, femp, ayuntamientos, podem, consistorios, bng, psoe
1st wave	Wuhan, conca, bei, ceoe, educamadrid, selectividad
2nd wave	Femp, anc, grec, jxcat, zbe
3rd wave	TSJC, ciutadans, afd, tv-3, ifema
MW general	Ministerio de sanidad, gobierno de coalición, pandemia del coronavirus, gobierno de pedro, consejo interterritorial, ejecutivo catalán, ejecutivo interterritorial, gobierno central, ayuntamiento de barcelona, ejecutivo de coalición, cc oo, departamento de salud, ejecutivo central, centro de coordinación, residencia de anciano, comunidad de Madrid, diputación permanente
1st Wave	Pacto de la Moncloa, batalla política, ejecutivo autónomo, política española, sesión de control, comisión de sanidad, decreto de estado, crisis de la covid-19
2nd Wave	Capital catalana, Ayuntamiento de Barcelona, Consejería de Sanidad, ejecutivo regional, ejecutivo madrileño, gobierno madrileño, fuente del gobierno, fuente del ejecutivo
3rd Wave	Bloque independentista, partido independentista, partido catalán, junta electoral, cola del hambre
*Covid 19 technolects*	
SW general	Coronavirus, covid-19, covid, virus, bioeasy, brote, sars-covid-2
2nd wave	Serológico
3rd wave	Pfizer

**Fig. 1:**
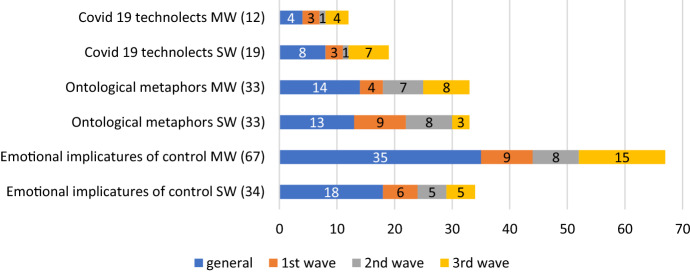
Manipulative devices in the English corpus

**Fig. 2:**
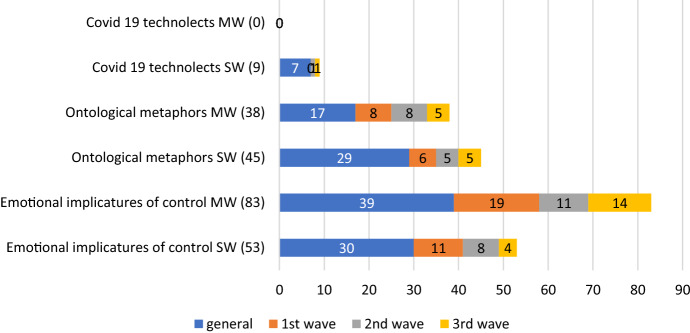
Manipulative devices in the Spanish corpus

Additionally, Sketch Engine has allowed us to get results in single words (SW) and multiwords (MW). Generally speaking, the latter make up for the majority (and most interesting part) of the data. Figures 1 and 2 show the incidence of manipulative devices in either subcorpus, and Table 14 and 15 minutely detail the findings in this area:

In view of the results above, some considerations need to be made. First, that, indeed, some terms which were not relevant in the general corpus appeared in the subsequent waves, highlighting the most pressing phenomena of the time. New expressions occur mainly in the groups of e-implicatures of control and ontological metaphors, and in both languages, whereas in the group of COVID19 technolects are scarce and, in the Spanish case, almost completely absent, if in the Third Wave of the English corpus they are as abundant as those in the general group, reflecting the appearance of new treatments and drugs. The most substantial part of our manipulative devices is made up by implicatures of control, which are inordinately abundant in the Spanish corpus, in relative terms as compared to the English corpus—136, against 103—and regarding the other lexical groups. This group was considered to be integrated by the mass of measures taken to make the virus visible and render the population aware of its dangers.

Words with very negative connotation such as ‘hospitalization’, ‘infection wave’, ‘death toll’, ‘death rate’, ‘national emergency’ and, in Spanish, *alarma* (‘emergency’), *funeraria* (‘funeral home’) *UCI* (‘intensive care unit’), *emergencia sanitaria* (‘health emergency’) and *zona de riesgo* (‘risk zone’) are examples of this. Also essential are the manoeuvres carried out by the State to combat the disease, which represent an important part of the data, and imply the part where the State takes it in their hands to control the population irretrievably. As instances of this group we can mention words some of which also appeared in our frequency list, such as ‘lockdown’, ‘isolation’, ‘mass testing’, ‘tracing’, ‘test and tracing’, ‘hotel quarantine’, ‘’circuit breaker’, and, in Spanish, *confinamiento* (‘lockdown’), *cuarentena* (‘quarantine’), *rastreo* (‘tracing’), *distanciamiento social* (‘social distancing’), *decreto de alarma* (‘emergency decree’), *plan de choque* (‘shock plan’) and *restricción de la movilidad* (‘restricted mobility’), just to give a few examples. At this point it would be fair to point out that we have made decisions when there was an overlapping of implicatures of control and technolects, such as ‘herd immunity’ or *prueba PCR* (‘PCR test’, or ‘polymerase chain reaction test’, in specialised lingo). Because these words have been made part of the popular jargon involving the virus, we found that they functioned more potently as implicatures than as signals of epistemic asymmetry.

Additionally, words like ‘coronavirus pandemic’, or *pandemia* (‘pandemic’) were very technical words at the beginning of the crisis, but we made the decision to include them as ontological metaphors, since they imply the reification of the virus, providing it with the qualities of a real phenomenon with a life of its own, such as a natural disaster, or as an enemy to combat. The result of both decisions might be the group of Covid-19 technolects in either corpus is smaller, especially in the Spanish one, but what is clear is that the British government and think tanks supplied the population with very technical lingo, which was not disseminated in the Spanish community.

In the English corpus names of laboratories and vaccines are common in metonymic forms (‘Moderna’, ‘Biontech’, ‘Pfizer’, etc.), but also chemical components and drugs, such as ‘hydroxychloroquine’, ‘glp1’ and ‘ivermectin’, as well as sophisticated methods for testing and diagnosis, such as the ‘Cygnus’, or ‘Cygnus exercise’, which is a simulation of an influenza contagious wave. With the exception of Pfizer, none of these terms appear in the Spanish corpus. This phenomenon is more or less replicated in the group of metaphorical ontologies.

In both corpora, political metonymies are common, mainly in the English corpus, such as ‘Whitehall’, ‘Westminster’, ‘Downing’ and *Moncloa* (the Spanish executive headquarters), but also ministerial offices, think tanks, and, mainly in the Spanish corpus, political parties, which abundantly predominate in it over the English corpus, in tune with our lexical frequency findings above. But it is also striking to note how in the English corpus a sizable number of abbreviations and acronyms are found, naming new committees created to fight the illness, such as NERVTAG (‘New and Emerging Respiratory Virus Threats Advisory Group’), CRG (‘Centre for Genomic Regulation’) and MHRA (‘medicines and health products regulatory agency’), being mere examples. The names of these are so alienating that they almost constitute examples of specialized jargon, defeating non-experts in their necessity to acquire information on the virus, and denote the plain superiority of the expert group.

If, finally, we observe ontological metaphors, we can see that they are ubiquitous in both corpora, but mainly in the Spanish one (with 83 metaphors, against 63 in the English corpus; they are aimed to give the impression that the State has contrived to create all kinds of ramifications in their need to keep the virus (and people) under control. However, in the Spanish panorama it appears as if central ministries, local governments and institutions, and their political pacts all over the country are getting ahold of the general attention, sometimes above matters of health, which is the case of the British corpus—where at least is the NHS and its various committees that take precedence. The politicization of the crisis is evident in Spain, the data seem to suggest. It does not help that the Government left matters of health in the hands of the different quasi-federal regions, the so-called Autonomous Communities, quite early in the process.

All in all, a significant number of manipulation devices for negative persuasion have been found, surpassing, as we will eventually demonstrate, every other group. If we take a look at the positive side of the corpus, however, we can see how scarce the results are for both corpora.

**Fig. 3:**
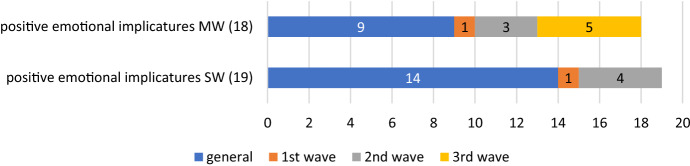
Positive persuasion emotional implicatures in the English corpus

**Fig. 4:**
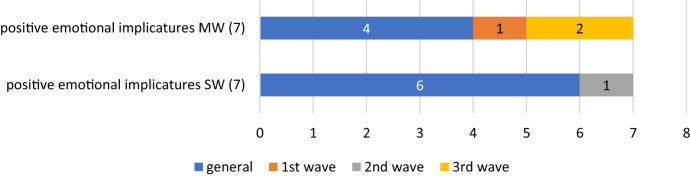
Positive persuasion emotional implicatures in the Spanish corpus

Figures 3 and 4 and Tables 16 and 17 show the results for either language.

**Table 16:** Examples of positive persuasion emotional implicatures in the English corpus

Positive emotional implicatures	
SW general	Furlough, covid-secure, reopen, easing
1st wave	Bailout
2nd wave	Hospitality, immunise, immunisation, edtech
MW general	Furlough scheme, universal credit, care home, free school, job support, green Recovery, job retention, support package, care staff
1st wave	Joint procurement
2nd wave	Business support, social care, economic recovery
3rd wave	Remote learning, negative test, festive period, winter grant, negative result

**Table 17:** Examples of positive persuasion emotional implicatures in the Spanish corpus

Positive emotional implicatures	
SW general	ERTE, eurobono, cogobernanza, mede, videoconferencia, mutualización
2nd wave	Teletrabajo
MW general	Fondo europeo, medida económica, fondo de recuperación, personal sanitario
1st wave	Servicio esencial
3rd wave	Ayuda directa, misión internacional

We have included in this group all those terms, words or concepts aimed, not at controlling the crisis, but at mitigating its consequences. Multiwords are, as in the previous taxonomies, more abundant in the English corpus, and marginally less so in the Spanish one than single words, as it also happened with e-implicatures of control and ontological metaphors.

New coinages and expressions take place in the subsequent waves of the English corpus, whereas there are few innovations in the Spanish case. Examples point to ways in which education and work conditions take new forms (‘edtech’, ‘remote learning’ in English, *videoconferencia*, *teletrabajo* in Spanish), and how the social network works—‘social care’, *servicio esencial* (‘essential service’)—but it is remarkable to see that most of the—scarce—expressions of a positive tone refer to the economy; they speak about economic measures aimed at alleviating the situation of those financially affected by the restrictions of movement, mainly with terms such as ‘furlough’ or *ERTE*, with identical meanings in each language.

Peculiar is to see the reliance of Spain on the EU for this—*fondo europeo* (‘EU fund’); *fondo de recuperación* (‘recovery fund’); *ayuda directa* (‘direct aid), and *eurobono* (‘Eurobond’)–, and the varied range of solutions provided by the British government (‘universal credit’, ‘business support’, ‘free school’, ‘bailout’, ‘support package’, etc.). Only in the English corpus a few of them refer to improvements in the health situation, such as ‘immunisation’ and ‘negative test/result’. All in all, and as it happens in our search for lexical frequencies, they do not compare to, and never offset, the range of negative persuasion lexis in the corpus.

## Conclusions

We started our work with one premise and two hypotheses; the first has neither been proven or disproven, since it constitutes our baseline of analysis, as ratified by previous studies [2, 3]: that the government uses its power through the mass media to impose restrictive courses of action in cases of crises, and that, specifically, the British and Spanish governments have used quality papers as harbingers of doom to impose lockdowns and other repressive measures during the COVID19 crisis. To work under such a premise, a representative and comparable ad hoc corpus of more than two million words was, then, compiled to ascertain that our work had a solid empirical basis.

The way in which this power has been deployed constituted our first and our second hypotheses, namely that the necessary persuasion to coerce or convince could have been accomplished through negative or positive devices, as shall be glossed over below. Because we even resorted to the socio-evolutionary theory of emotions, or AST (affect spectrum theory, as in 6) to better illustrate how power, persuasion and manipulation are connected to our basic and most sophisticated emotions.

Our first hypothesis, we think, has been proved: that in order to exert pressure over the population, the government through the mass media used authoritative verbal devices, proper of agonic, hierarchical societies, over the three waves of the epidemic. Enthymemes in the shape of e-implicatures were deployed to control, pressure, impose, almost in equal measure, in the news discourse of either country y—if they were especially present in the Spanish case—and each of the three waves saw new incorporations of these words that reflected the *status quo* of each of the stages in the crisis.

Ontological metaphors were exploited to substantiate abstract conceptualizations, and they appeared singularly in each of the waves: the Spanish government did this through the chorus of the country’s political parties and (some of them, very troublesome) autonomous governments, caring very little about the people they had to serve.

Very technical terms were wielded to sustain gnoseological asymmetry: how—mainly—the British government, through the public health authorities and authorised expert voices, endeavoured to impress citizens with epidemiological terminology in what we deem to be ‘interdiscursive appropriation’ [20]: politicians making use of expert voices to persuade and keep citizens in awe of the unknown, and rely on who they know.

But there was another side to our argument, and a second hypothesis: that, in the context of more evolved, democratic, hedonic societies, it is not enough to coerce, but authorities have to use persuasion to convince the public that incredibly harsh measures are good for them and are keeping them alive: this would lead us to the analysis of the so-called ‘hooray’ or positive words, verbs guaranteeing discretionary powers to the people and words of the affect paradigm indicating positive or prosocial emotions. Words like ‘optimism’ and *optimismo* were used in negative contexts, as warnings to the citizens not to get too careless in their abeyance to orders. Others, like ‘clear’—*claro*–, qualify the crispness of the State’s orders to their people; Nouns like ‘trust’, ‘hope’, ‘confidence’, and in Spanish *calma*, *confianza* and *seguridad* always refer to the self-propaganda that the State uses to confer the suitability of their orders and instructions. Sadly, other positive, more enthusing words were much scarcer in either corpus, indicating that in the sombre spectre of a crisis, the hope for salvation is much more restricted. The words ‘immunisation’ and ‘vaccine’ (which the British press, as spokesmen of the government, duly use, if the Spanish press does feebly, we have proved) are the only bright lines in the horizon.

At the time we write, though, our future is still stifled with the prospect of new outbreaks and future curfews. The Delta variant and its new version, Delta Plus, has found its way all over the United Kingdom and, in Spain, young people, still mostly unvaccinated because of a less successful campaign than in Britain, rebel and conspire to drink in droves and without protection. And on top of it all, the State has learnt their way to rob people of their liberties for their own good, and, in our view, they have liked the way it worked. Let our study be a warning that we, linguists, and semioticians, are aware of it.

## Supplementary Information

Below is the link to the electronic supplementary material.

Supplementary file 1 (PDF 800 kb)

## Footnotes

1 The first front line of the COVID crisis, according to Wagner et al. [4], is constituted by health workers and the health community at large, whereas the second front line is comprised of liberal practitioners, who had to impose measures to protect themselves and their patients.

2 In Spain, they took the form of a series of decree-laws subsequently ratified by Parliament, in the shape of the so-called Covid-19 Health Crisis Management Measures (*Medidas de gestión de la crisis sanitaria Covid 19,* in https://cutt.ly/emdEN34), in tune with EU legislation. The United Kingdom, in contrast, passed a comprehensive statute, the Coronavirus Act, 2020 (in https://cutt.ly/gmdE2ow).

3 As Charteris-Black reminds us [10: p. xiii], ‘deliberative’ language is used to establish political decisions, while ‘epideictic’ language is used to achieve consensus.

4 Available at https://tinyurl.com/yz4j7hk7.

5 UK domain represents 7.9% of the whole corpus (https://www.sketchengine.eu/ententen-english-corpus/) and the European Spanish domain accounts for 49.32% (https://www.sketchengine.eu/estenten-spanish-corpus/).

6 At the time of writing this paper, Hancock had just resigned from his post as Health Secretary on account of a scandal by a breach of sanitary measures.

7 Illa was the Secretary of Health during the three subsequent waves; at the time of writing the paper, he had (unsuccessfully) abandoned his post to become a candidate in the Catalonian elections.

8 Even if ‘positive’ has an upbeat denotation, its connotation in the corpus is negative since it refers mostly to coronavirus positive diagnoses.

9 Ídem supra.
